# From placenta to the foetus: a systematic review of in vitro models of stress- and inflammation-induced depression in pregnancy

**DOI:** 10.1038/s41380-024-02866-1

**Published:** 2024-12-05

**Authors:** Madeline Kirkpatrick, Gargi Mandal, Ismail Elhadidy, Nicole Mariani, Kristi Priestley, Carmine M. Pariante, Alessandra Borsini

**Affiliations:** Department of Psychological Medicine, Stress, Psychiatry and Immunology Laboratory, Institute of Psychiatry, Psychology and Neuroscience, King’s College, London, UK

**Keywords:** Cell biology, Molecular biology, Neuroscience

## Abstract

**Background:**

Depression in pregnancy can increase vulnerability for psychiatric disorders in the offspring, likely via the transfer of heightened maternal cortisol and cytokines to the *in-utero* environment. However, the precise cellular and molecular mechanisms, are largely unclear. Animal studies can represent this complex pathophysiology at a systemic level but are expensive and ethically challenging. While simpler, in vitro models offer high-throughput opportunities. Therefore, this systematic review integrates findings of in vitro models relevant to depression in pregnancy, to generate novel hypotheses and targets for intervention.

**Methods:**

The systematic analysis covered studies investigating glucocorticoid or cytokine challenges on placental or foetal neural progenitor cells (NPCs), with or without co-treatment with sex hormones.

**Results:**

Of the 50 included studies, 11 used placental cells and 39 NPCs; surprisingly, only one used a combination of oestrogen and cortisol, and no study combined placental cells and NPCs. In placental cells, cortisol or cytokines decreased nutrient transporter expression and steroidogenic enzyme activity, and increased cytokine production. NPCs exhibited decreases in proliferation and differentiation, via specific molecular pathways, namely, inhibition of hedgehog signalling and activation of kynurenine pathway. In these cells, studies also highlighted epigenetic priming of stress and inflammatory pathways.

**Conclusions:**

Overall, results suggest that stress and inflammation not only detrimentally impact placental regulation of nutrients and hormones to the foetus, but also activate downstream pathways through increased inflammation in the placenta, ultimately eliciting adverse effects on foetal neurogenesis. Future research should investigate how sex hormones regulate these mechanisms, with the aim of developing targeted therapeutic approaches for depression in pregnancy.

## Introduction

According to the developmental origins of health and disease hypothesis, environmental stressors in the intrauterine environment during critical periods of foetal development can predispose the individual to illness in later life [1]. Depression in pregnancy, affecting approximately 20% of women globally [2, 3], induces alterations in maternal physiology that can impact foetal neurodevelopment during these vulnerable periods, increasing the risk for psychiatric and behavioural disorders [4–11]. Indeed, about 40–50% of offspring exposed to depression in pregnancy experience psychopathology in later life [12]. Therefore, uncovering the mechanisms through which maternal depression can confer poorer offspring mental health is an important avenue for research and may allow for identification of new therapeutic targets.

The heightened risk of adverse outcomes in offspring associated with depression in pregnancy remains evident even when controlling for the influence of shared genetics [13], disrupted maternal care [7, 14], and other risk factors associated with maternal mental health, such as smoking [9] and an unhealthy diet [15]. This highlights that depression in pregnancy directly alters the biology of the uterine environment to affect foetal neurodevelopment. However, the conclusive molecular and cellular mechanisms are unknown. Despite this, in vivo animal models and human clinical data have highlighted the involvement of changes in epigenetics, neurogenesis, synaptogenesis and priming of stress and inflammatory pathways [11, 16–19].

One proposed mechanism through which depression in pregnancy may influence the foetal brain is through increased maternal activity of the hypothalamic-pituitary-adrenal axis (HPA) and subsequent increased production of the glucocorticoid hormone, cortisol [20]. In both prenatal depression and depression outside of pregnancy, cortisol levels have been demonstrated to be elevated [21]. While glucocorticoids are essential for foetal development, chronically high levels can be detrimental. For example, elevations of cortisol are known to both decrease and increase proinflammatory cytokines, based on the experimental conditions, as well as to diminish brain-derived neurotrophic factor (BDNF) synthesis, and increase synaptic glutamate levels, all of which are implicated in the development of psychopathology [22–25]. Exposure of the foetus to high levels of cortisol during development may also alter HPA axis function, increasing susceptibility to later depression [12, 21].

Additionally, cortisol has been demonstrated to decrease hippocampal neurogenesis [26] and increase apoptosis [22]. Normally, the placenta protects the foetus from high levels of cortisol through conversion to its inactive state, cortisone, using the enzyme 11 β-hydroxysteroid-dehydrogenase type 2 (11β-HSD2). However, studies suggest that depression in pregnancy can decrease the function of this enzyme, increasing the exposure of the foetus to cortisol [27–30]. Indeed, clinical studies in humans have highlighted that offspring of prenatally depressed mothers show increased cortisol levels and a dysregulated HPA axis response [21, 31, 32].

In addition to HPA axis dysregulation, previous evidence suggests maternal immune activation (MIA) as a contributing factor for foetal neurodevelopment alterations, and consequent intergenerational transmission of psychiatric risk [21, 33–36]. MIA, induced experimentally by administering immune activators to pregnant dams [37] or studied clinically in the context of infection during pregnancy, predisposes the offspring to an increased risk for neurodevelopmental disorders [38, 39]. Depression in pregnancy is also associated with an increase in pro-inflammatory cytokines such as tumour necrosis factor-alpha (TNF-α), interleukin-6 (IL-6), IL-1β and IL-17 [21, 40–45], prompting the question of whether the heightened inflammatory milieu in depression in pregnancy can affect the developing foetus in a similar manner as MIA. Indeed, cytokines can cross the placenta and elicit effects on foetal neurodevelopment, with high inflammatory levels persisting in the offspring during and long after the postnatal period [36, 42].

While the potential involvement of stress and inflammation on placental function in foetal programming remains not fully understood, it is likely that the placenta is a key mediator in the mechanisms of intergenerational transmission [46]. It may be that the maternal psychological state leads to peripheral stress-related changes that affect the placenta’s activity, which in turn affects the foetus in a detrimental manner [47–49].

A large body of studies focussing on placental or foetal neurodevelopment in depression in pregnancy, either in vivo or in humans, have been previously extensively reviewed [18, 35, 46, 48, 50]. However, due to the complexity of the maternal-foetal interface and the plethora of physiological changes occurring during pregnancy, in vivo or clinical studies cannot give exact mechanistic insight on both cellular and molecular changes. Moreover, animal and clinical studies are expensive, invasive and ethically challenging, especially for a high-throughput hypothesis-generating approach. In vitro models provide simpler but very controlled conditions that allow to test a myriad of mechanisms and experimental manipulations, usually involving one or two cell types combined, which might be particularly relevant in this context because of the obvious role of stress-induced placental changes upon foetal brain cells. Thus, the aim of this systematic review is to provide, for the first time, a comprehensive understanding of the effect of stress and inflammation on both placental and foetal brain cells responses in vitro models – models that we would argue are relevant to our understanding of depression in pregnancy. A systematic review was chosen over a narrative review to minimize bias, enhance reproducibility, and provide a more rigorous, structured synthesis of evidence relevant to glucocorticoid and cytokine impacts on placental and fetal neural progenitor cells (NPCs).

## Methods

A systematic review was conducted on studies using in vitro cell models to investigate the impact of prenatal stress and depression on foetal development. The three online databases used for this review were Web of Science, Ovid and PubMed. To obtain all relevant papers, we employed several keywords and Boolean operators in the advanced search query option of the stated databases. The search strategy was as follows: (“antenatal depression” OR “depression in pregnancy” OR “maternal stress” OR “stress in pregnancy” OR “early life stress” OR “maternal immune activation” OR “prenatal stress”) AND (“in vitro” OR “cell model*” OR “cell culture” OR “placenta cell*” OR “trophoblast cell” OR “foetal neural progenitor*” OR “progenitor cell*” OR “neural progenitor*” OR “hippocampal progenitor”). A supplementary manual search of references was conducted in order to screen for additional publications. Following this, references from all three databases were collected, and duplicates were removed. An initial search was conducted in December 2023, and updated on the 15th of April 2024.

Subsequently, screening for eligibility was performed, firstly in titles and abstracts and then papers carried forward were subject to a full-text analysis. Studies were eligible for inclusion if they used an in vitro experiment, if the model involved an insult with molecules thought to be present in depression in pregnancy (specifically glucocorticoids and cytokines), and if they looked at the effect of this insult on developing neural or trophoblast cells. There were no restrictions on year of publication. Papers were excluded from the review if they were review papers, book chapters, conference papers, commentaries, case reports, or not fully accessible. The included studies examine the effects of elevated glucocorticoids and cytokines on the development and function of either placental or foetal NPCs. The Preferred Reporting Items for Systematic Reviews and Meta-Analysis (PRISMA) were followed. It is important to acknowledge that the use of immortalized progenitor cell lines in a few of the identified studies may not always have been intended to directly model brain development however we included them here due to the progenitor phenotype of the cells.

**Fig. 1:**
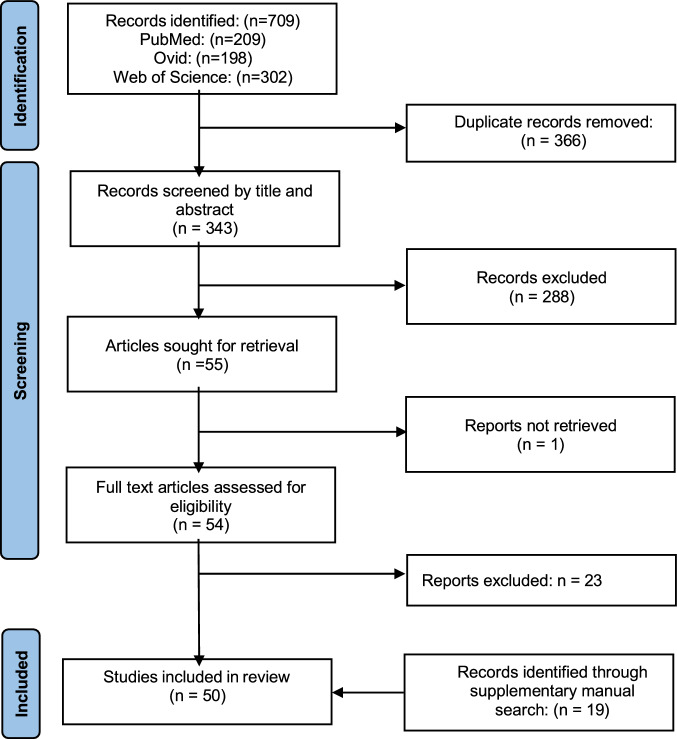
PRISMA flow diagram for study inclusion protocol. This diagram shows the systematic process followed by the authors in order to include papers captured by the literature search.

Fig. 1 shows the PRISMA flow diagram for study inclusion protocol. Of the Fifty included studies, eleven were conducted in placental cells, and thirty-nine in NPCs. Studies were further divided into those which used a stress insult, and those which used an inflammatory insult. Study characteristics summarising the challenge used, cell type, outcomes and mechanisms can be seen in Table 1.

**Table 1:** **a**. Summary of studies investigating the effects of stress and inflammatory insults on placental cells in vitro. **b**. Summary of studies investigating the effects of stress and inflammatory insults on foetal neural progenitor cells in vitro.

Studies using placental cells										
Challenge used	Length of exposure	Cell type	Species	Outcomes					Mechanisms	Ref
				Transport	Apoptosis	Placental hormonogenesis	Inflammatory /stress response	Genetic/ Epigenetic changes		
**Stress insult**										
Dexamethasone (10^−^^6^ mol/L) or Cortisol (10^−^^8^ mol/L)	72 h	Placental villous explants, isolated syncytiotrophoblast and cytotrophoblast and Bewo cells	Human	↑BCRP					Syncytiotrophoblast cells are the response site for GR-mediated ↑BCRP	[51]
Cortisol (10, 100 and 1000 nM)	12 h	BeWo and JEG-3	Human					↓DEPTOR	DEPTOR is a modulator of mTOR signalling – could have consequences for placenta development.	[52]
Cortisol (10, 100 and 1000 nM)	12 h	BeWo and JEG-3	Human					↑GAS5	GAS5 is a ss-ncRNA which can repress the GR by acting as a decoy GRE	[53]
Cortisol (20 ng/ml)	24 – 48 h	Sw.71 trophoblast cells	Human				↓CSF2 and CSF3		↓CSF2 and CSF3 – cortisol increases DNA methylation	[54]
Cortisol (0–1200 nM)	24 h	BeWo	Human	↓GLUT1, GLUT2, LDLR, ABCA1, TAUT and LAT1		↓11β-HSD2		↑C/EBPα and Egr1 ↓Sp1	↓11β-HSD2: Cortisol increased enrichment of Egr1 in the 11β-HSD2 promotor ↓Glucose, lipid, cholesterol and aa transporter expression was due to ↓11β-HSD2	[55]
Cortisol (500 ng/ml)	3 days	BeWo	Human	↓EV secretion						[56]
**Inflammatory insult**										
IL-17a (100 ng)	1 h	BeWo cells and primary trophoblast cells	Human	↑IL-17a transport			↑IL-17RA		IL-17a accumulates in and passes through placenta by binding to IL-17RA	[57]
IL-1β, IL-6 or TNF-α (10 ng/ml)	24 h	Primary placental villous explants	Human			↓11β-HSD2 activity		No changes in 11β-HSD2 mRNA	↓1βB-HSD2 activity mediated by ↑Ca^2+^ and ↓AC activity acting through post transcriptional mechanisms.	[60]
TNF-α (10 ng/ml)	24 h	Primary trophoblasts	Human			↑CYP24A1 ↓CYP19, ↓HSD3β1, ↓hCG	↑IL-6, ↑IFN-γ		TNF-α ↑calcitriol degrading enzyme CYP24A1. Calcitriol inhibits TNF-dependent inflammation.	[59]
TNF (10 pg/ml – 10 ng/ml)	48 h	BeWo	Human		↑	↑CYP11A1 and CYP19A1	↑TNF and Nf-kB	↑ERW-1	High TNF levels (1–10 ng/mL) ↑inflammatory response, physiological TNF concentrations ↑cell viability	[58]
AICAR (0.5 or 1.0 mM) or rapamycin (50 or 100 nM)	30 min or 3 h	JAR	Human	↓EAAT and ASCT1					↓EAAT2 – EAAT2 normally extracts glutamate from fetal circulation, increased glutamate can cause neurotoxicity.	[61]

Studies using neural progenitor cells											
Challenge used	Length of exposure (stage of maturation)*	Cell type	Species	Outcomes						Mechanisms	Ref
				Proliferation	Apoptosis	Gliogenesis	Neurogenesis	Inflammatory /stress response	Genetic/ Epigenetic changes		
**Stress insult (human cells)**											
Cortisol ( < 100 nM) or aldosterone (1 nM –1 μM)	3 days (P) and/or 7 days (D)	Fetal hippocampal progenitors (HPC0A07/03 C)	Human	↑		↑	↓		↑Notch and Hes signalling ↓Hedgehog	↑Proliferation: MR activation (100 nM cort) ↓Proliferation: GR activation (1 µM cort) - ↓TGF-B-SMAD2/3 and ↑FOX03A signalling. ↑Astrogliogenesis: MR activation - ↑Notch and Hes signalling ↓Neurogenesis: MR and GR activation - ↓Hedgehog signalling.	[26]
Cortisol ( > 1 µM) or dexamethasone (10 nM – 5 μM)				↓		=	↓		↓TGFB-SMAD2/3 and ↑FOX03A ↓Hedgehog		
Cortisol (100 µM)	3 days (P) and/or 7 days (D)	Fetal hippocampal progenitors (HPC0A07/03 C)	Human	↓			↓		↑SGK1 ↓Hedgehog	↓Proliferation and neurogenesis: GR-induced ↑SGK1, which ↓Hedgehog signalling and ↑GR phosphorylation and nuclear translocation	[62]
Cortisol (100 µM)	3 days (P)	Fetal hippocampal progenitors (HPC0A07/03 C)	Human						↓miR-125b-1-3p Modulation of 1145 DEGs, 7 of which are modulated by miR-125b-1-3p.	↓miR-125b-1-3p may have long lasting effects on future neurodevelopment and neuroinflammation.	[63]
Cortisol (100 µM)	24 h (P)	Fetal hippocampal progenitors (HPC0A07/03 C)	Human	↓					↑Fox01 and SGK1	↓Proliferation: GR ↑Fox01 and SGK1, inhibition of Fox01 prevents ↑SGK1	[64]
Cortisol (100 µM)	3 days (P)	Fetal hippocampal progenitors (HPC0A07/03 C)	Human						↓miR-19a and miR-19b in proliferating and mature neurons	Cortisol ↓miR-19, which may alter neurodevelopmental and immune/inflammatory processes.	[65]
Cortisol (100 µM)	3 days (P)	Fetal hippocampal progenitors (HIP-009)	Human						↓miR-20b-5p and miR-29c-3p and ↑NR3C1, FKBP5 and Fox01 in proliferating and mature neurons	Cortisol ↓miR-20b-5p and miR-29c-3p, leading to long-term ↑NR3C1, FKBP5 and Fox01	[72]
a) Cortisol (100 mM)	3 days then 3 days (P) and/or 7 days (D)	Fetal hippocampal progenitors (HPC0A07/03 C)	Human	↓	↑		↓		EPA and DHA: regulate NRF2, STAT3, IFN, and IL-1 DHA: regulates CREB	EPA and DHA prevent cortisol-induced ↓neurogenesis by regulating pathways involved in oxidative stress and immune response	[69]
b) EPA (10 mM) or DHA (10 mM) then cortisol (100 mM)				↑ (compared to (a))	↓ (compared to (a))		↑ (compared to (a))				
NSC-derived astrocytes - cortisol (1 µM).	48 h (post-D)	iPSC derived NSCs and NSC-derived astrocytes	Human		↑	↑Neurotoxic A1- astrocytes			↓FgFr1 in neurons and ↓FGF2 in astrocytes	↓Neurogenesis: ↑A1-like reactive astrocytes have ↓release of FGF2 and ↓neuronal FGFR1 leading to ↓Neuroligin1 and reduced glutamatergic pathway-dependent neurogenesis	[17]
NPCs - ACM	5 days (D)						↓				
Dexamethasone (1 µM)	24 h (P) or 4 days (D)	Primary fetal neural progenitor cells	Human	↓		↑	↓		↑Dkk1 ↓B-catenin ↓Cyclin D1	↑Dkk1: GR binds to the Dkk1 promotor Dkk1 induces changes in proliferation, neurogenesis and Gliogenesis by inhibiting canonical Wnt-signalling, leading to ↓cyclin D	[73]
Dexamethasone (1 µM)	3 days (P) or 10 days (P + D)	Fetal hippocampal progenitors (HPC0A07/03 C)	Human				↓ = after washout	↑ Transcriptional response to secondary GC challenge	Widespread gene expression changes, majority not maintained after DEX removal. Long lasting DNA methylation changes. ↑TET1 and ↑UHRF1	Changes in DNA methylation: ↑TET1 and ↑UHRF1 ↑Response to secondary GC challenge: DNA methylation primes transcripts to an altered transcriptional response on subsequent GR activation	[66]
Dexamethasone (1 µM)	10 days	Fetal hippocampal progenitors (HPC0A07/03 C)	Human	↓			↓			Sertraline prevents dexamethasone-induced ↓ neurogenesis	[67]
Dexamethasone (1 µM) + sertraline (1 µM)				↑			↑				
Corticosterone (25-400 µM)	24 h	SH-5Y5Y neuroblastoma cells	Human					↑IL-6 and IL-10	↓LC3B-II	↑IL-6 and IL-10: Decrease autophagy function enhances immune activation	[70]
IL-1β (10 ng/mL)	24 h per treatment (P)	Fetal hippocampal progenitors (HPC0A07/03 C)	Human					↑IL-6		Inflammatory priming: DEX pre-treatment activates GR, leading to upregulation of inflammatory pathways	[68]
IL-1β (10 ng/mL) + DEX (1–100 nM)								↓IL-6	↑NLRP6		
Pre DEX + IL-1β											
								↑↑IL-6	↑NLRP6		
Corticosterone (B) (0.6 mmol/l)	1 h, 6 h, 12 h or 24 h	SH-5Y5Y neuroblastoma cells	Human						↓Tll-1 mRNA	↓Tll-1 mRNA: GR binds to the proximal GRE within the Tll-1 promotor Tll-1 may function as a potentiator of neurogenesis through regulating BMP signalling.	[71]
**Stress insult (rodent cells)**											
Corticosterone (2–40 µM)	3 days (D)	Primary fetal hippocampal progenitors	Rat	↓	>20 µM ↑		↓		↓ cyclin D2 ↓IGF-1 and IGF-1R ↓NeuroD, BDNF and NT-3, ↓NR1	↓Proliferation: CORT: ↓cyclin D2, DEX: ↑p21 ↓Neurogenesis: MR activation, ↓CREB, BDNF and NR1	[74]
Dexamethasone (5 µM)				↓			=		↑p21		
Corticosterone (0.001–10 µM)	3 days (P) and/or 7 days (D)	Embryonic neural stem/ progenitor cells	Rat	=		↓	↓			↓Differentiation: suppression of ERK and PI3K/Akt signalling	[77]
Corticosterone (100–400 µM)	24 h (D)	PC12 cells	Rat		↑				↑PSD-95	↑PSD-95: corticosterone-induced increase in autophagy and ↓ cell viability.	[79]
Corticosterone (1 and 10 µg/ml) and/or glutamate (10 and 100 µg/ml)	1–-3 days (D)	Organotypic hippocampal cultures	Rat		24 hr GLU + CORT: ↑ 72 hr CORT +/or GLUT: ↑			↑TNF-α	↑Bax 72 hr (All treatment): ↓BDNF and *Ngf*	↑Apoptosis: ↑TNF-α	[22]
Corticosterone (0, 50, 400 or 800 ng/ml) and/or E2 (0, 20, or 40 pg/ml)	24 h	HT-22 hippocampal cells	Mouse					E2 + CORT: ↑Fkbp5 ↓PPid		↑Fkbp5 (suppressor of GR translocation) and ↓Ppid (facilitator of GR translocation) may lead to suppression of GR translocation	[80]
Dexamethasone and corticosterone (1 µM)	3 days (P) or 5 days (D)	Embryonic NSCs	Rat	↓			No change			↓Proliferation: GR activation and by ubiquitination and proteasome-mediated degradation of cyclin D	[78]
Aldosterone (1 × 10^−^^8^ –1 × 10^−^^7^ M)	3 days (P & D)	Primary fetal hippocampal cells	Rat	↑			↑			↑Proliferation and neurogenesis: activation of the MR ↓Proliferation and neurogenesis: activation of the GR	[75]
Dexamethasone (1 × 10^−^^7^–1 × 10^−^^6^ M)				↓			↓				
CRH (1 × 10^−^^9 ^M – 1 × 10^−^^5 ^M)	16 h	Primary fetal hippocampal cells	Rat		↑				↑GRP78, XBP-1 splicing and CHOP	CRH activated IRE1/ASK/JNK cascade of ER stress, which contributed to increased apoptosis	[76]
**Inflammatory insult (human cells)**											
IL-12 (20 pg/ml)	1 day (P)	Fetal hippocampal progenitors (HPC0A07/03 C)	Human	↓	↑					↓Proliferation and neurogenesis: JAK/STAT pathway	[81]
IL-13 (25 pg/ml)	3 days (D)				↑		↓				
IL-1β (10 ng/mL)	3 days (P) and/or 7 days (D)	Fetal hippocampal progenitors (HPC0A07/03 C	Human	↑			↓		↑STAT1 ↑IDO ↑KMO and ↑KYNU	↓Neurogenesis: Activation of kynurenine pathway and increased production of neurotoxic metabolites	[82]
**a)** IL-1β (10 ng/ml)	10 days (P and D)	Fetal hippocampal progenitors (HPC0A07/03 C)	Human				↓		↑IDO ↑KMO and ↑KYNU	ω-3 PUFAs prevent IL-1β-induced ↓ neurogenesis by preventing neurotoxic kynurenine pathway activation.	[83]
**b)** IL-1β (10 ng/ml) + EPA/DHA (10 μM)							↑ (compared to a)		↓ IDO (compared to a)		
IL-6 (0.1 pg/ml – 100 ng/ml)	3 or 24 h	iPSC-derived microglia and NPCs	Human					NPCs: no change MGL: ↑IL-6, IL-10 and JMJD3	MGL: ↑IRF8, REL, HSPA1A/B and OXTR	No response in NPCs is due to the absence of IL-6R expression and (s)IL-6Ra secretion. In MGLs: IL-6 activates STAT3-signalling, leading to NFkB pathway activation and changes to ROS, cell adhesion, cytokine secretion and TNFRSF signalling.	[87]
Hyper IL-6 (s-IL6R covalently bound to IL-6) (25 ng/ml)	5 to 10 days (D)	iPSC-derived forebrain organoids	Human					↑MHCI	↑NR2F1 and STAT-3	Trans-IL-6 activates STAT3-signalling ↑Number of RGCs, which had highest number of DEGs	[89]
Hyper IL-6 (s-IL6R covalently bound to IL-6) (25 ng/ml)	5 days (D14-D19)	iPSC-derived NPCs	Human					↑P-STAT3	↑NR2F1	Trans-IL-6 activates STAT3-signalling	[88]
	5 days (D21-D26)							↑STAT3 ↑P-STAT3			
**a)** IL-6 (5 pg/ml or 50,000 pg/ml)	10 days (P and D)	Fetal hippocampal progenitors (HPC0A07/03 C)	Human				=			↓Neurogenesis: IL-1β and IL-6 ↑IL-8 Very high ( > 50,000 pg/ml IL-6) rescues ↓neurogenesis caused by IL-1β by ↓ release of IL-8	[84]
**b)** IL-6 (50, 500 or 5000 pg/ml)							↓				
**c)** IL-6 (5 pg/ml) with IL-1β (10 pg/ml)					↑		↓	↑IL-1B, IL-6, IL-8 and IL-13			
**d)** IL-6 (50000 pg/ml) with IL-1β (10 pg/ml)					↑		↑ (compared to **(c)**)	↓IL-8 (compared to **(c)**)			
TNF (50 or 250 pg/ml)	8 days	iPSC -derived cerebral organoids	Human	↓		↑	↓		↓FGFR1	Changes in FGFR signalling alter neurogenesis and gliogenesis	[90]
IFN-α (500 pg/mL and 5000 pg/mL)	3 days (P) and 7 days (D)	Fetal hippocampal progenitors (HPC0A07/03 C)	Human		↑		↓	↑IL-6	↑STAT1 ↑ISG15 ↑USP18 ↑UBA7, ↑UBE2L6 and ↑HERC5	↓Neurogenesis: ISG15-dependent STAT1 activation, and STAT mediated ↑UBA7, ↑UBE2L6 and ↑HERC5 ↑Apoptosis: IL-6 dependent STAT1 activation and STAT1-mediated down-regulation of AQP4	[85]
IFN-γ (25 ng/ml)	5 days (NPC stage) and/or 2 days (neuronal stage)	iPSC-derived NPCs and neurons	Human					↑IFN-γ response to second hit	1834 DEGs – enriched for MHCI genes. ↑GRM2 and ↓LHX6. Repeated exposure induced more DEGs than single treatment	IFN-γ induced transcriptional priming increased response to second hit. ↑MHC1 genes: mediated by ↑PML nuclear bodies ↑PML nuclear bodies and ↑MHC1 genes increase neurite outgrowth through B2M	[91]
IFN-γ (10 ng/ml)	24 h	BE [2]-M17 Neural progenitor cells	Human					Dysregulation of genes involved in stress and immune response networks	↓lnc-RNA GOMAFU ↑GOMAFU suppressed DEGs in the IFN-y pathway	↓GOMAFU contributes to increased IFN-signalling and alters neuronal responses to inflammatory insults	[92]
**a)** IL-1β (10000 pg/ml) IL-6 (50 pg/ml) or IFN-α (50,000 pg/ml)	48 hr (D)	Fetal hippocampal progenitors (HPC0A07/03 C)	Human		↑		IL-1β: ↓ IL-6: ↓ IFN-α: ↓			LOX- and CYP450-derived EPA/DHA metabolites prevent cytokine-induced ↓ neurogenesis and ↑ apoptosis, by preventing cytokine-induced ↑STAT1, NF-kB and ↓AQP4	[86]
**b)** EPA/DHA (10 μM) + IL-1β (10000 pg/ml), IL-6 (50 pg/ml) or IFN-α (50,000 pg/ml)	48 hr + 6 or 48 hr (D)				↓ (compared to **(a))**		↑ (compared to **(a))**				
**Inflammatory insult (rodent cells)**											
IL-1β (10 ng/ml)	2 h	Hippocampal progenitors	Rat	↓	=		↓			↓Proliferation: IL-1β action on IL-1RI receptors reduces cell cycle, does not increase cell death. Involves Nf-kB Ikk pathway and ↓cyclin D	[93]
IL-1β (10 ng/ml)	4 days (P) or 7 days (D)	Embryonic hippocampal NPCs	Rat	↓	↑	↑	↓		↑IL-1R mRNA	Changes in proliferation and differentiation mediated by IL-1R1 signalling	[94]
IL-1α or IL-1β (50 or 500 pg/mL)	12 – 48 h	Primary neurospheres	Mouse	↓						↓Proliferation: reduce Trb2+ NP entry into the S phase of cell cycle, by suppressing increase in CyclinD1 and Cyclin B	[95]
IL-17A (0.01–100 ng/mL)	48 h (P) or 6 days (D)	Primary OPC	Mouse	↓		↑		↑Cytokine mRNA		IL-17A activates ERK1/2 MAPK pathway in OPCS	[96]
IFN-a (100–1000 IU/ml)	24 h	HT-22 hippocampal cells	Mouse					↓DEX-induced GR binding to GRE	Co-immunoprecipitation of STAT5 and GR	↓GR function: activation of Jak-STAT and nuclear STAT5-GR protein-protein interactions	[97]

*BCRP* Breast Cancer Resistant Protein, *GR* Glucocorticoid Receptor, *mTOR* Mammalian Target of Rapamycin, *GAS5* Growth Arrest Specific Transcript-5, *ss-ncRNA* single-strand non-coding RNA, *GRE* Glucocorticoid Response Element, *CSF* Colony-Stimulating Factor, *GLUT* Glucose Transporter, *LDLR* Low Density Lipoprotein Receptor, *ABCA1* ATP-binding Cassette Subfamily A member 1, *TAUT* Taurine Transporter, *LAT1* Large Amino Acid Transporter 1, *11β-HSD2* 11-beta-hydroxysteriod-dehydrogenase type 2, *C/EBPα* CCAAT/enhancer binding protein-alpha, *Egr1*, Early growth response protein 1, *Sp1* Specific protein 1, *EV* Extracellular Vesicle, *IL* Interleukin; *IL-17RA* Interleukin-17A Recepto,*TNF-α* Tumour Necrosis Factor-alpha, *mRNA*messenger RNA, *AC* Adenylyl Cyclase, *CYP24A1* Cytochrome P450 family 24 subfamily A member 1,*CYP19* Cytochrome P450 aromatase, *HSD3β1* Hydroxy-Delta-5-Steroid Dehydrogenase, 3 Beta- And Steroid Delta-Isomerase 1, *hCG* human Choriogonadotropin, *IFN-γ* Interferon-gamma, *CYP11A1* cytochrome P450 family 11, *Nf-κβ* Nuclear factor kappa-light-chain-enhancer, *EAAT* Excitatory Amino Acid Transporter, *ASCT1* neutral Amino Acid Transporter, *Hes* Hairy/Enhancer of split, *MR* Mineralocorticoid Receptor, *TGF-β* Transforming Growth Factor-beta, *SGK1* Serum/Glucocorticoid Related Kinase 1, *EPA* Eicosapentaenoic acid, *DHA* Docosahexaenoic acid, *NRF2* Nuclear factor erythroid 2-related factor 2, *STAT* Signal Transducer and Activator of Transcription, *CREB* cAMP Response Element Binding Protein, *NSC* Neural Stem Cell, *iPSC* induced Pluripotent Stem Cells, *NPCs* Neural Progenitor Cells, *ACM* Astrocyte Conditioned Media, *FGFR1* Fibroblast growth factor receptor 1, *Dkk1* Dikkopf, *DEX* Dexamethasone, *TET1* Ten Eleven Translocation 1, *UHRF1* Ubiquitin-like containing PHD and RING Fingers Domain 1, *LC3B-II* LC3-phosphatidylethanolamine Conjugate, *GC* glucocorticoid, *NLRP6* NOD-like Receptor Family Pyrin domain containing 6, *Tll-1* Tolloid 1, *BMP* bone morphogenic protein, *IGF-1* insulin-like growth factor-1, *IGF-1R* IGF-1 receptor, *NeuroD* Neurogenic Differentiation factor, *BDNF* Brain Derived Neurotrophic Factor, *NT-3* Neurotrophin-3, *NR1* Nuclear Receptor 1,*CORT* Cortisol, *ERK* Extracellular Related Kinase, *PI3K* Phosphatidylinositol 3-kinase, *Akt* protein kinase B, *PSD-95* Postsynaptic Density Protein 95,*GLU*, Glutamate, *Ngf* Nerve growth factor, *E2* Estradiol, *Fkbp5* FK506 binding protein 5, *Ppid* co-chaperone peptidylprolyl isomerase D, *GRP78* 78-kDa Glucose-Regulated Protein, *XBP-1* X-box Binding Protein 1, *CHOP* C/EBP Homologous Protein, *CRH* Corticotrophin Releasing Hormone, *IRE1* Inositol-requiring enzyme 1, *ASK* Apoptosis Signal-regulating kinase*, JNK* Jun N-terminal kinase*, ER* Endoplasmic Reticulum, *JAK* Janus Kinase, *IDO* Indolamine-2,3-Dioxygenase, *KMO* Kynurenine 3-Monooxygenase, *KYNU* Kynureninase, *ω-3 PUFAs* Omega-3 Polyunsaturated Fatty Acids, *MGL* Microglia, *IRF8* Interferon Regulatory Factor 8, *HSPA1A/B* Heat Shock Protein A1A/B, *OXTR* Oxytocin Receptor, *ROS* Reactive Oxygen Species, *TNFRSF* Tumour Necrosis Factor Receptor Subfamily, *MHCI* Major Histocompatibility Complex, *NR2F1* Nuclear Receptor Subfamily 2 group F, *RGCs* Radial Glial Cells, *DEG* Differentially Expressed Gene, *UBA7* Ubiquitin-like Modifier Activating Enzyme 7, *UBE2L6* Ubiquitin Conjugating Enzyme E2 L6, *HERC5* HECT And RLD Domain Containing E3 Ubiquitin Protein Ligase 5, *AQP4* Aquaporin 4,*MAPK* Mitogen Activated Protein Kinase, *OPC* Oligodendrocyte Progenitor Cell.

* For stage of maturation, (P) refers to proliferating cells and (D) refers to differentiated cells.

## Results

### The effect of glucocorticoids on placental cells

Six studies assessed glucocorticoid effects on human placental cells [51–56]. Among these, three showed that cortisol treatment induced placental transport changes, including reduced extracellular vesicle (EV) release [56], increased breast cancer resistant protein (BCRP) expression [51], and reduced expression of glucose, lipid, cholesterol and amino acid transporters, which are important for transferring nutrients from the maternal circulation to the foetus [55]. Additionally, in BeWo cells, a trophoblast cell line derived from human choriocarcinoma, cortisol treatment decreased expression of the enzyme 11β-HSD2, which is involved in the conversion of cortisol to the inactive cortisone [55].

The other three studies reported genetic or epigenetic changes in placental cells following exposure to glucocorticoid [52–54]. In one, cortisol treatment downregulated the expression of DEP domain containing mTOR-interacting protein (DEPTOR), a modulator of mTOR signalling [52]. Additionally, cortisol was shown to repress glucocorticoid receptor (GR) function by upregulating the non-coding RNA (ncRNA) growth arrest-specific transcript 5 (GAS5) [53], and to inhibit the expression of colony-stimulating factor 2 (CSF-2) and CSF-3, which are important for placental development [54].

Overall, these studies show that glucocorticoids impact placental cells by decreasing transport mechanisms, decreasing cortisol de-activation, and inhibiting the expression of molecules important for placental cell development and function, such as DEPTOR and CSF-2/3.

### The effect of inflammation on placental cells

Five studies investigated the effect of inflammatory cytokines, again, on human placental cells. Among them, four studies directly treated placental cells with cytokines [57–60], while one study used an indirect method to model immune activation [61]. One demonstrated that IL-17A is able to cross the placenta by binding to IL-17RA [57], and two studies demonstrated the upregulation of distinct inflammatory pathways in response to TNF-α including the production of IL-6 and interferon-gamma (IFN-γ), as well as the activation of the Nuclear factor kappa-light-chain-enhancer (Nf-kB) pathway [58, 59]. In particular, in primary trophoblast cells, TNF-α decreased the expression of cytochrome P450 aromatase (CYP19) [59], an enzyme involved in oestrogen biosynthesis. Conversely, in syncytialized BeWo cells, TNF-α increased expression of this enzyme [58]. In another study, treatment of primary villous explants with IL-6, IL-1β or TNF-α decreased the activity of 11β-HSD2, via reduction of adenylyl cyclase, which is an enzyme involved in the production of cyclic adenosine monophosphate (cAMP) from adenosine trisphosphate (ATP) [60].

The fifth study used an indirect method to model cytokine signalling in JAR cells, another placental cell line derived from human choriocarcinoma [61]. JAR cells were treated with the mammalian target of rapamycin complex 1 (mTORC1) inhibitor rapamycin and the AMP-activated protein kinase (AMPK) activator 5-aminoimidazole-4-carboxamide ribonucleoside (AICAr) in vitro to mimic the in vivo effects of MIA on the placenta. As a result, protein expression of excitatory amino acid transporter 2 (EAAT2) and alanine/serine/cysteine/threonine transporter 1 (ASCT1) were significantly reduced, which suggests that activation of the maternal immune system can decrease transport of essential amino acids to the foetus.

Overall, these studies show that inflammatory cytokines impact placental cells by upregulating inflammatory pathways, decreasing steroidogenic enzyme activity, and decreasing the expression of amino acid transporters.

### The effect of glucocorticoids on NPCs

Twenty-two studies examined the effects of glucocorticoids on NPCs. Of these, fourteen used human cells: nine (all from our group) used the foetal hippocampal progenitor cell (HPC) line (HPC0A07/03 C) [26, 62–69], two used the SH-5Y5Y neuroblastoma cell line [70, 71], one used the HIP-009 HPC line [72], one used induced pluripotent stem cell (iPSC)-derived NPCs [17], and one used primary foetal NPCs [73]. Eight studies used rodent NPCs, of which three used primary foetal HPCs [74–76], two used embryonic neural stem cells (NSCs) [77, 78], one used organotypic hippocampal cultures [22], and two used cell lines: the PC12 cells [79] and HT-22 hippocampal cells [80]. These twenty-one studies looked at a variety of outcomes, including proliferation, neurogenesis, inflammatory pathways, and epigenetic changes, which we will describe separately.

### The effect of glucocorticoids on NPC proliferation and differentiation

Nine studies, six using human cells and three using rodent cells, reported changes in proliferation following glucocorticoid exposure. In human NPCs, treatment with cortisol or the synthetic GR agonist dexamethasone, at a concentration known to induce a GR activation ( > 1 µM), decreased cell proliferation [26, 62, 64, 67, 69, 73]. However, in a study from our group, treatment with cortisol at a concentration of <100 nM induced a mineralocorticoid receptor (MR) activation and subsequently increased cell proliferation [26]. Similarly, in rodent NPCs, corticosterone (rodent equivalent of cortisol) and dexamethasone treatment at a concentration greater than 1 µM decreased cell proliferation [74, 75, 78], while treatment with aldosterone (a specific MR agonist), increased cell proliferation [75].

Ten studies, seven using human cells and three using rodent cells, reported changes in NPC neurogenesis. In human NPCs, studies from our group have consistently reported that glucocorticoids decrease neurogenesis in our human HPC line [26, 62, 66, 67, 69]. Interestingly, one study from our group demonstrated that, while dexamethasone did decrease neuronal differentiation, this effect did not persist following washout [66]. Additionally, in two other studies from our group, both the antidepressant sertraline [67] and omega-3 polyunsaturated fatty acids (ω-3-PUFAs) [69], prevented glucocorticoid-induced decreases in neurogenesis in our human HPCs. Two additional studies reported that treatment with glucocorticoid decreased neuronal differentiation in human NPCs [17, 73]. Accordingly, a study in rodent NPCs showed that glucocorticoid treatment decreased neurogenesis [77]. However, two studies in rat HPCs found opposite results. The first study showed that aldosterone increased neurogenesis [75], and the other one, using dexamethasone, found no effect [74].

Four studies, three using human cells and one using rodent cells, found changes in gliogenesis in response to glucocorticoids. In human NPCs, glucocorticoid treatment increased astrogliogenesis [17, 26, 73]. Our study in human HPCs also showed that cortisol (100 nM) or aldosterone (1 nM-1 μM) increased astrocytic differentiation, while high cortisol (100 µM) or dexamethasone (10 nM–5 μM) had no effect [26]. In human NSC-derived astrocytes and in astrocyte conditioned media (ACM)-treated NPCs, treatment with 1 µM cortisol increased differentiation of NSCs towards A1-like reactive astrocytes, but ACM treatment decreased neuronal differentiation of NPCs [17]. However, in rodent cells, corticosterone decreased glial differentiation [77].

Overall, studies show that high glucocorticoid concentrations impact NPCs by decreasing proliferation and neuronal differentiation and increasing differentiation into glial cell types, albeit results are different when using lower concentrations that are not relevant to stress.

### Changes in molecular signalling pathways following glucocorticoids exposure

Among the aforementioned studies, and one previously not mentioned [80], eleven also investigated molecular signalling pathways elicited by glucocorticoids to induce changes in proliferation and differentiation.

Three studies from our group reported molecular mechanisms leading to changes in neurogenesis in our human HPC line [26, 62, 64]. One demonstrated the involvement of GR-mediated upregulation of serum/glucocorticoid related kinase 1 (SGK1) in inhibiting hedgehog signalling and enhancing GR phosphorylation for nuclear translocation [62]. An inhibitor of SGK1 prevented reductions in neurogenesis, confirming its role in mediating these outcomes. In another, cortisol treatment upregulated FoxO1, with inhibition of FoxO1 preventing GR-induced elevations in SGK1 [64]. Similarly, in HIP-009 HPCs, FoxO1 was upregulated by 100 μM cortisol treatment [72]. Finally, another study from our group showed that glucocorticoid treatment was associated with a decrease in transforming growth factor-beta (TGFβ)-SMAD2/3 and hedgehog signalling, with hedgehog signalling confirmed to mediate decreases in neurogenesis [26]. Further, MR activation led to increased Notch- and Hairy/Enhancer of Split signalling, which has been previously shown to cause a shift from neurogenesis to astrogliogenesis during neurodevelopment [26]. Another group, also using human NPCs, showed that glucocorticoids decreased cyclin D via GR-mediated upregulation of Dikkopf1 (DKK1) and subsequent inhibition of Wnt-signalling, a promoter of neurogenesis and cell proliferation, eventually leading to reduced neurogenesis [73].

Consistent with these last results, two studies using rodent NPCs also showed that glucocorticoids decreased cyclin D levels to reduce proliferation [74, 78]. In one, this stemmed from enhanced cyclin D degradation via the ubiquitin proteasome system [78]. Moreover, in rat NPCs, corticosterone treatment inhibited BDNF [74] and phosphatidylinositol 3-kinase (PI3K) / protein kinase B (Akt) signalling [77], leading to decreased neurogenesis. In primary rat foetal HPCs, treatment with corticotrophin-releasing hormone (CRH), a stress-induced peptide that acts both upstream of glucocorticoid secretion and directly on target tissues, induced the IRE1/ASK1/JNK cascade, leading to increased apoptosis and endoplasmic reticulum (ER) stress [76]. In one study, corticosterone increased autophagy, leading to reductions in cell viability [79]. In a study in HT-22 mouse hippocampal cells, corticosterone increased the expression of Fkbp5, a negative regulator of GR signalling. This is believed to be a regulatory mechanism whereby increased levels of cortisol prevent prolonged GR translocation. Additionally, exposure to corticosterone plus oestradiol potentiated the increase in Fkbp5 expression [80], indicating that oestradiol might be a inhibitor of GR action. This last study is the only one that used sex hormones in this context.

While most of these mechanistic studies investigated only neuronal cells, one study using ACM-treated human NPCs showed that glucocorticoids decreased astrocytic release of fibroblast growth factor 2 (FGF2), resulting in impaired glutamatergic synapse formation and neurogenesis [17].

Overall, these studies show that glucocorticoids affect NPC proliferation and differentiation via several mechanisms, including a reduction of cell cycle proteins, inhibition of hedgehog signalling, and reduced growth factor signalling. Additionally, oestradiol can regulate GR activity and may play a role in protecting the fetus from the detrimental effects of glucocorticoids.

### Changes in inflammatory pathways following glucocorticoids exposure

Three studies did not report on cellular outcomes but rather showed increases in inflammatory signalling in response to glucocorticoids. In human cells, treatment of SH-5Y5Y neuroblastoma cells with corticosterone decreased autophagy function, which led to increased IL-6 and IL-10 production, although the mechanisms are unclear [70]. A study from our group showed that, in human HPCs, co-treatment with dexamethasone reduced IL-1β-induced IL-6 production [68]. Interestingly, pre-treatment with dexamethasone followed by IL-1β *increased* IL-6 production further than IL-1β treatment alone. This was due to GR-dependent inflammatory priming and upregulation of Nod-like receptor 6 (NLRP6), a receptor which participates in inflammasome activation and regulation of NF-κB and mitogen-activated protein kinase (MAPK) signalling. In rat organotypic hippocampal cultures, treatment with glutamate and corticosterone increased the production of TNF-α, resulting in enhanced apoptosis [22].

Overall, these studies show that glucocorticoid treatment upregulates inflammatory pathways in NPCs.

### Epigenetic changes following glucocorticoids exposure

Four studies, one of which has been previously mentioned [66], reported epigenetic changes following glucocorticoid exposure [63, 65, 66, 72]. Three of these studies were from our group and used the human HPC line, HPC0A07/03 C [63, 65, 66]. In the first one, dexamethasone treatment induced long-lasting DNA methylation changes which primed transcripts to a greater response upon a second dexamethasone challenge [66]. The other two studies reported changes in micro-RNA (miRNA) levels [63, 65], with both demonstrating a cortisol-induced reduction in miR-19 in human HPCs. When cortisol was used during proliferation, decreased miR-19 persisted following differentiation, suggesting the effects of this reduction are long-lasting [65]. Cortisol induced the differential expression of 208 miRNAs in HPCs, 137 of which were upregulated and 71 downregulated [63]. However, only downregulation of miR-125b-1-3p was a common finding between clinical and animal models. Gene targeting and pathway analysis revealed both miR-19 and miR-125b-1-3p target pathways involved in the inflammatory and immune response, and in neurodevelopment [63, 65]. The final study exploring epigenetic changes used the human HPC line, HIP-009. Cells were treated with cortisol during proliferation, which led to an upregulation of NR3C1, FoxO1 and FKBP5. These expression changes were maintained after 21 days of differentiation, which was associated with a downregulation of the miRNAs miR-20b-5p and miR-29c-3p [72].

Overall, these studies show that glucocorticoids induce epigenetic changes in NPCs which target pathways involved in neurodevelopment and inflammation.

### The effect of inflammation on NPCs

Eighteen studies analysed the effect of inflammatory cytokines on NPCs. Thirteen used human cells, of which seven used HPC0A07/03 C HPCs [68, 81–86], five used iPSC-derived NPCs [87–91] and one used BE [2]-M17 NPCs [92]. Additionally, five studies used rodent cells, four of which used primary NPCs [93–96] and one used HT-22 hippocampal cells [97]. As in the section above, we discuss each outcome (cellular, molecular, epigenetic) separately.

### The effect of cytokines on NPC proliferation and differentiation

Eleven studies, seven using human NPCs and four using rodent cells, reported changes in proliferation and/or differentiation following cytokine exposure. Studies from our group showed that treatment of the human HPC line with IL-12 decreased cell proliferation [81], while IL-1β increased proliferation [83]. Furthermore, we have also shown in these cells that treatment with IL-13 [81], IL-1β [82, 83, 86], IL-6 [86], and IFN-α [85] decreased neuronal differentiation. Interestingly, a study from our group further identified that co-treatment with IL-6 (5 pg/ml) and IL-1β (10 ng/ml) decreased neurogenesis, whereas IL-6 at (50,000 pg/ml) prevented IL-1β-induced reductions in neurogenesis [84]. In contrast, treatment with IL-6 alone at 5 or 50,000 pg/ml caused no changes, whereas intermediate concentrations (50, 500 and 5000 pg/ml) decreased neurogenesis. In another study in human iPSC-derived cerebral organoids, TNF-α treatment decreased neuronal differentiation and increased glial differentiation [90]. In rodent HPCs, IL-1β decreased cell proliferation [93–95] and decreased neuronal differentiation [93, 94], whereas both IL-17A [96] and IL-1β [94] increased glial differentiation.

Overall, these studies show that cytokine treatment in NPCs decrease neuronal differentiation and increases glial differentiation, although these effects are concentration dependent.

### Changes in molecular signalling pathways following cytokines exposure

Among the aforementioned studies using human cells, five of them also investigated additional molecular signalling pathways elicited by cytokines to induce changes in proliferation and differentiation. Of these, four studies are from our group. The first study showed that IL-1β treatment induced signal transducer and activator of transcription 1 (STAT1) activation and increased enzymes of the neurotoxic arm of the kynurenine pathway, including upregulation of indolamine-2,3-dioxygenase (IDO) and kynurenine 3-monooxygenase (KMO) and subsequent increased production of kynurenine metabolites in HPCs supernatant [82]. Importantly, inhibition of IDO, the first enzyme of this pathway, prevented IL-1β-induced reductions in neurogenesis, demonstrating its role in mediating this outcome. Two other studies from our group have also shown that ω-3-PUFAs treatment rescues IL-1β-induced reduction of HPC neurogenesis and increase in apoptosis by inhibiting neurotoxic kynurenine components [83, 86]. ω-3-PUFAs are considered potential antidepressants and have shown anti-inflammatory action [98]. Further, in our human HPC line, IFN-α decreased neuronal differentiation by ISG15-dependent STAT1 activation and subsequent upregulation of the E1 (UBA7), E2 (UBE2L6) and E3 (HERC5) enzymes regulating ISG15 ISGylation of proteins involved in neuronal differentiation [85]. Additionally, IFN-α [85] and IL-6 [86] were shown to increase apoptosis via STAT-1 mediated downregulation of aquaporin 4 (AQP4), with ω-3-PUFAs being able to prevent these detrimental effects [86].

Finally, in one study in human iPSC-derived cerebral organoids, TNF-α decreased proliferation, increased gliogenesis and decreased neuronal differentiation through a reduction in fibroblast growth factor receptor 1 (FGFR1) signalling [90]

Overall, these studies show that, similarly to glucocorticoids, the mechanisms elicited by cytokines to induce changes in proliferation and differentiation are vast. Mechanisms reported include activation of neurotoxic kynurenine pathways, decreased growth factor signalling, and upregulation of ISGylation.

### Genetic and epigenetic changes following cytokine exposure

Five studies in human NPCs reported either epigenetic changes or described genetic changes alongside long-term effects of inflammatory challenges that are likely to be related to epigenetic changes. All these studies were in human NPCs [87–89, 91, 92].

Among them, two used Hyper-IL-6, a chimeric protein which allows for trans-signalling as the soluble IL-6 receptor is covalently bound to IL-6 [88, 89]. One study in dorsal forebrain organoids (DFOs) [89] and another in iPSC-derived NPCs [88] showed that treatment with Hyper-IL-6 resulted in STAT3 activation and upregulation of nuclear receptor subfamily 2 group F1 (NR2F1). In DFOs, this resulted in the upregulation of genes associated with the innate immune response, such as major histocompatibility complex 1 (MHC1) gene members. Similarly, in two studies, exposure of NPCs to IFN-γ [91, 92] induced transcriptional changes in stress and inflammatory gene networks, including upregulation of the MHC1 complex, and an increased response to a second IFN-γ hit [91]. Additionally, one study in BE [2]-M17 cells showed that IFN-γ treatment downregulated the long ncRNA GOMAFU, resulting in a de-repression of GOMAFU-supressed genes of the IFN-γ signalling pathway and subsequently increasing inflammatory signalling [92]. One study in HT-22 cells demonstrated that IFN-α treatment reduced dexamethasone-induced binding of GR to the glucocorticoid response element (GRE) through activation of JAK/STAT5 [97], as STAT5 prevented GR-GRE binding.

Overall, these studies show that treatment of NPCs with inflammatory cytokines induces persistent, likely genetic and epigenetic, changes which increase inflammatory signalling pathways in developing neuronal cells. Additionally, studies show that inflammatory cytokines can interact with GR signalling mechanisms.

## Discussion

**Fig. 2:**
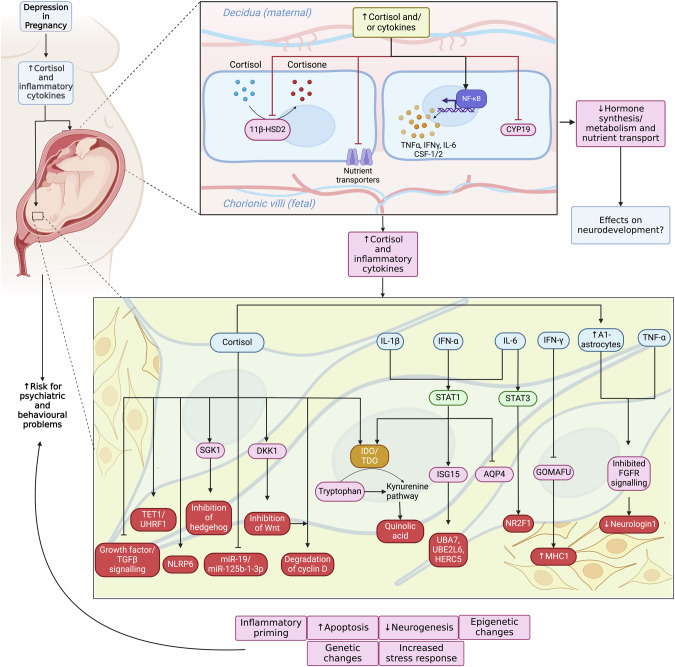
Schematic summary of the molecular and cellular mechanisms elicited by heightened cortisol and inflammatory cytokines in placental and NPCs. Depression in pregnancy increases cortisol and inflammatory cytokines in the maternal circulation. These act on the placenta to decrease cortisol de-activation, decrease nutrient transport, increase inflammatory cytokine expression and alter steroid hormone synthesis. This highlights that the effects of heightened cortisol and cytokines on the placenta are greater than their simple transport across the barrier. Cortisol and cytokines also directly affect fetal brain development, leading to increased apoptosis, decreased neurogenesis, and stress and inflammatory priming. These outcomes occur via multiple mechanisms and increase risk for psychiatric and behavioural disorders in the affected offspring. 11β-HSD2, 11 β-hydroxysteriod-dehydrogenase type 2; IL-1β, interleukin 1 beta; IFN-α interferon alpha; TNF-α, tumour necrosis factor alpha; STAT, signal transducer and activator of transcription; SGK1, serum/glucocorticoid related kinase 1; DKK1, Dikkopf 1; IDO, indoleamine 2,3 dioxygenase; TDO, tryptophan-2,3-dioxygenase; ISG15, interferon stimulated gene 15; AQP4, aquaporin 4; NRF2, nuclear receptor subfamily 2 group F1; MHC1, major histocompatibility complex; FGFR, fibroblast growth factor receptor.

To our knowledge, this is the first systematic review which comprehensively summarises and integrates findings from in vitro models relevant to depression in pregnancy, that is, using glucocorticoids and inflammatory challenges in placental and foetal brain cells. In placental cells, most studies demonstrate that cortisol or cytokines decrease nutrient transporter expression and steroidogenic enzyme activity and increase the production of inflammatory cytokines. In foetal brain cells, most of the studies report a decrease in NPCs proliferation and differentiation, with inhibited hedgehog signalling, decreased cell cycle proteins, and kynurenine pathway activation being the mechanisms mostly involved in the observed cellular outcomes. In these cells, studies additionally describe a priming of stress and inflammatory pathways through epigenetic mechanisms, such as decreases in non-coding RNAs and changes in DNA methylation. Overall, results suggest that stress and inflammation not only have a detrimental impact on the way the placenta regulates the availability of nutrients and hormones to the foetus, but also activate, through increased inflammation in the placenta, downstream pathways ultimately eliciting detrimental effects on foetal brain neurogenesis (Fig. 2).

### The placenta as a key mediator of foetal programming

A critical strength of this review is the incorporation of models studying the effects of depression-associated factors on placental cells. The placenta elicits mechanisms to shield the foetus from adverse maternal environments [46] and thus studying how cortisol and cytokines impact the foetal brain without first questioning their effects on placental function would be missing a vital step in the pathophysiological mechanisms of foetal programming [46].

The most used cell model found in the literature was the BeWo cell line. Originally isolated from human choriocarcinoma, BeWo cells display morphological properties common to placental trophoblast cells, and synthesize placenta-specific hormones such as human chorionic gonadotrophin [99]. Exposure of these cells to both cortisol [55] and a model of MIA [61] decreases expression of transporters for glucose, amino acids, lipids and cholesterol. This is of particular interest given the role that maternal nutrient insufficiencies play in offspring cognitive development [100]. Clinical studies show that the level of placental lipids (including ω-3-PUFAs) is inversely correlated with prenatal depressive symptoms, and in turn, reduced placental lipids predict poorer offspring socio-emotional outcomes [101–103]. However, in these clinical studies it is not possible to separate the effect of depression-associated biological factors from changes in the maternal diet caused by depression [104]. Results from the reported in vitro models highlight that depression in pregnancy may alter nutrient transport independently from the maternal nutritional status, due to the direct effect of cortisol and cytokines on placental transporter proteins. Along the same lines, cortisol also attenuates the release of EVs from placental cells [56], and such EVs are important for foetal neurodevelopment [105].

### The effects of glucocorticoids and inflammation on the placenta

Several studies reviewed here demonstrate alterations in placental enzymes important for hormone synthesis in response to glucocorticoids and cytokines [55, 58–60]. Indeed, clinical studies have highlighted that changes in placental hormone synthesis can affect the developing foetal brain [106]. Of note, these studies lack the investigation of enzymes involved in sex steroid synthesis (such as CYP19 (aromatase) and 3βHSD), prompting the need for further research, particularly considering the demonstrated neuroprotective effects of oestradiol and allopregnanolone [106]. Furthermore, serotonin receptor activation increases placental CYP19 activity [107, 108], suggesting that decreased serotonin levels due to depression in pregnancy may alter placental oestrogen production [109]. Additionally, in an in vitro co-culture model of the feto-placental unit (to model feto-placental steroidogenesis [110]), treatment of BeWo cells with serotonin reuptake inhibitors (SRIs) induced CYP10 activity via the serotonin receptor signalling pathway [108, 111]. However, an active SRI metabolite, norfluoxetine, decreased placental oestrogen production [111]. These results highlight that antidepressant use during pregnancy can additionally affect placental oestrogen production. Importantly, both cortisol [55] and TNF-α [60] reduce activity of 11β-HSD2, potentially increasing the exposure of the foetus to cortisol. Indeed, both clinical studies [29, 30] and animal models [28] have demonstrated an effect of maternal depression on placental 11β-HSD2. The presented studies support this association while also proposing a mechanistic role for cortisol and cytokines.

Previous clinical evidence has suggested that cord blood concentrations of cytokines are greater as a result of prenatal stress [112]. One study reported here shows that cytokines (specifically IL-17A) are capable of crossing the placental barrier to the foetal circulation [57], and while this is supported by findings from some animal models [113], others demonstrate that not all cytokines possess this ability [114]. However, in addition to the direct transport of cytokines, studies also measure changes in placental expression of inflammatory signalling molecules following stress and inflammatory insults [54, 58, 59]. Clinical studies [115] and animal models [49] support this association by demonstrating increased expression of immune response genes in placentas from stressed and depressed pregnancies. This suggests that depression in pregnancy may cause the placenta to produce more cytokines than the foetus would be expected to be exposed to from the maternal circulation directly. Indeed, clinical studies have highlighted that placental inflammatory genes can predict depressive symptoms in the offspring during adulthood [116].

### From the placenta to the foetal brain

These downstream stress- and inflammation-related pathways, activated through the placenta, may in turn affect the neurodevelopment of the foetal brain, and ultimately cause long-term consequences. While the models reviewed here cannot directly test how placental changes affect foetal brain development, they can help generate hypotheses about potential causal mechanisms. For instance, from the placental cell studies identified, we hypothesize that stress and inflammation in the placenta may reduce the supply of neuroprotective factors like nutrients and reproductive hormones to the foetal brain while increasing exposure to cortisol and cytokines. The main reported effect of both cortisol and cytokines on NPCs is changes in cell proliferation and differentiation. Studies consistently show that increased NPC proliferation in response to low cortisol concentrations ( < 100 nM) is due to activation of the MR, while decreased proliferation caused by higher concentrations of cortisol ( > 1 μM) is due to GR activation [26]. Similarly, while high concentrations of IL-6 potentiate IL-1β-induced reductions in neurogenesis, supraphysiological concentrations of IL-6 have the reverse effect [84]. Few human studies have documented cortisol and cytokine concentrations in cord blood from depression in pregnancy, and results suggest the presence of a wide range of concentrations [117–119]. Nevertheless, in our review, one study did report that treatment of primary trophoblast cells with TNF-α induces secretion of IL-6 at a concentration of 500 pg/ml [59], which is within the range of concentrations shown by our group to be detrimental to HPC neurogenesis in vitro [84]. However, the studies included do not report on the concentrations of cortisol secreted by placental cells in response to insults. Therefore, to ensure the translatability of models, it is essential to further validate the specific concentrations of cortisol and cytokines released into the foetal circulation.

Studies reported here also consistently demonstrate that glucocorticoid and inflammatory insults decrease neuronal differentiation, irrespective of their concentration. With reference to foetal programming, decreased neurogenesis during development may entail difficulties in regulating the stress response due to disrupted hippocampal circuits. Indeed, clinical studies support this association by demonstrating that maternal stress and depression are associated with reductions in hippocampal volume [120, 121] and increased cortisol reactivity to stress in offspring [21]. Additionally, most studies reported here agree that glucocorticoid and inflammatory insults increase gliogenesis [17, 26, 73, 90, 94]. However, in vivo animal models suggest contrasting results [122]. Interestingly, one study included in this review shows that cortisol treatment induces an A1-like reactive astrocyte phenotype, which impacts neurogenesis by decreasing neurotrophic support [17]. This suggests that alterations in glial cell function by stress and inflammatory insults may also detrimentally affect neuronal development. Further, this also highlights the importance of considering the effects of neighbouring cells in any in vitro experimental model, prompting the use of co-culture systems and organoid cultures which can mimic a more complex cellular environment.

A series of studies from our group using the HPC0A07/03 C line can be integrated to decipher the molecular signalling pathways leading to cortisol-induced decreases in hippocampal neurogenesis [26, 62, 64]. Synthesising findings from these studies reveals that activation of the GR increases expression of FoxO1, which increases SGK1 transcription. Activation of SGK1, in turn, increases GR phosphorylation, promoting its nuclear translocation, and inhibits hedgehog signalling, resulting in decreased neurogenesis. However, reported mechanisms of cortisol-induced changes in neurogenesis are vast, as can be seen in Fig. 2. This is also true for cytokines, where changes in neurogenesis are underscored by increased neurotoxic kynurenine activity [82, 83], STAT1 signalling pathways [81, 85] and reduced cyclin D [93, 95]. These multiple mechanisms leading to the same biological outcome constitute a difficulty in identifying effective therapeutic targets and critical points for intervention.

Despite this, studies do report the use of interventions which prevent reductions in neurogenesis, such as ω-3-PUFAs [69, 83, 86] and antidepressants [67]. However, the use of antidepressants during pregnancy should be limited to clinically-significant cases; while generally considered safe [123], research has shown that some can negatively impact foetal brain development due to dysregulation of the neurotrophic role of serotonin, an area of urgent research [124].

The protective effects of ω-3-PUFAs are particularly interesting, given they are decreased in the placenta in both a clinical study [101] and in placental cells treated with cortisol [55], suggesting that depression in pregnancy may starve the foetus of the protective mechanisms of placental ω-3-PUFAs. Similarly, oestradiol reduces GR translocation [80], highlighting that hormone levels may additionally regulate the detrimental effects of cortisol.

### Long-term outcomes on the foetal brain following glucocorticoid and inflammatory insults

While the immediate impact of glucocorticoid and inflammatory insults on neurogenesis is evident, it is also important to consider the enduring genetic and epigenetic changes that may potentiate an increased risk for psychiatric disorders by ‘priming’ stress and inflammatory pathways. Indeed, under the “multiple-hit model” of intergenerational transmission, it is hypothesised that, while the *i*n utero environment can prime an increased risk for psychiatric disorder, disease arises when other adverse events occur in later life [19, 125]. One study reported in this review highlights that the cortisol-induced reduction in neurogenesis is transient in nature, but alterations in DNA methylation are persistent, and these induce a heightened response upon a subsequent stress insult [66]. This suggests that exposure to cortisol during development can increase the set point of the stress response, increasing vulnerability to stress exposure in later life.

Similarly, other studies included in this review, employing both glucocorticoids and cytokines, indicate a priming of the inflammatory response [68, 91, 126], with several showing the involvement of decreased ncRNAs in the process [63, 65, 92]. Interestingly, one study reported here shows that pre-treatment of cells with cortisol before IL-1β treatment potentiates the inflammatory response, due to neuroinflammatory priming [68]. Indeed, clinical studies and animal models demonstrate that offspring exposed to depression in pregnancy experience a heightened immune response to inflammatory stimuli [127], with one longitudinal study even demonstrating that this persisted at 25 years of age [128]. This evidence all highlights the potential for preventative interventions targeting inflammatory pathways, such as ω-3-PUFA supplementation [83, 86, 129, 130], in offspring of depressed mothers who will be more likely to develop mental health disorders in later life.

### Linking in vitro results to in vivo and clinical data

The correlation of in vitro findings from the reported models into in vivo and clinical contexts is essential for understanding the broader implications and relevance of these results. In line with the findings of the reported in vitro studies, animal models exploring the impact of prenatal depression on the placenta report decreases in placental 11β-HSD2 [28], increased placental inflammation [49] and reduced placental lipids [101]. Evidence from the reported studies highlighting the same outcomes from in vitro treatment with glucocorticoids and cytokines highlights that these insults may play a significant role in driving these changes in vivo. In turn, clinical studies have demonstrated associations between maternal depression and increased levels of cortisol [117] and inflammatory cytokines [112], as well as decreased lipids [101, 103], within the foetal circulation. With reference to the changes occurring within foetal NPCs, several of the studies reviewed here performed additional animal [63–65, 70, 71, 75, 79, 95] or human [63, 64, 72] analysis alongside in vitro work to confirm translatability of their findings. For instance, Fujioka and colleagues found that mild prenatal stress, which mimics lower cortisol levels, increased hippocampal proliferation in rat pups, while severe stress impaired hippocampal neurogenesis [75]. These findings are consistent with their own results in rat hippocampal cells, as well as with human in vitro data from our group [26]. Additionally, studies examining epigenetic changes in NPCs exposed to stress and inflammatory insults have found correlations with the epigenetic alterations observed in animals and humans exposed to prenatal stress [63, 65, 72].

### Limitations and the future of in vitro models of depression in pregnancy

While this review was robust in its design, there are some limitations which need consideration. Firstly, this review incorporated studies using both human and animal in vitro models. The results reported here demonstrate that species differences do exist in NPC responses to cortisol and cytokines, and thus future studies should focus on replicating findings also in human cells in order to ensure the translatability of models. Secondly, while the in vitro models discussed here give critical insight into the molecular signalling pathways elicited by cortisol and inflammatory cytokines, this is not necessarily the full picture. In the case of depression in pregnancy, the various protective and risk factors involved in foetal programming are likely to create a complicated picture of multiple interacting signalling pathways. Additionally, the studies included in this review do not account for the effect of postnatal factors, which likely also contribute in creating a continuous adverse or protective environment which modulate the risk of the offspring developing negative mental health outcomes. Further, several clinical studies have highlighted the presence of sex differences in foetal programming [55, 131, 132], something which was not considered in any of the in vitro models presented. This further highlights the potential role for sex hormones in regulating the effects of stress and inflammation on fetal brain development. However, while these limitations exist, this review offers an opportunity for future studies to further investigate the identified mechanisms, and ultimately understand how multiple signalling pathways interact at the placental and foetal level to determine risk or resilience.

Advancing the future of in vitro models for depression in pregnancy will require integrating new technologies and refining existing methodologies to better mimic the complex prenatal environment. For example, a model to study placental steroidogenesis has been developed, whereby placental cell lines are co-cultured with human adrenocortical carcinoma cells (to represent the fetal adrenal gland) [110]. Such a model considers the steroidogenic interactions between the placenta and fetus. Additionally, newly developed dynamic in vivo-like in vitro models such as organ-on-a-chip offer a promising approach in their ability to better recapitulate the in vivo environment and can be used to investigate barrier and transport functions of the placenta [133] (for a review on organ-on-chip models of the maternal-fetal interface see [134]). This method has also been used to model neurodevelopment within a brain organoid-on-a-chip system [135]. However, to determine if changes induced in the placenta by an adverse maternal environment produce causal downstream mechanisms influencing fetal neurodevelopment, future models will need to begin to combine both cell lines. In this sense, static in vitro models may offer a more promising approach, and have the additional benefit of simplicity and high-throughput hypothesis testing. For example, co-culture of placental cells and NSCs has been performed using transwell models, whereby placental cells are cultured in the apical compartment, and NSCs are cultured in the basal compartment. Alternatively, placenta-conditioned media can be collected from the basal compartment of the transwell and used to directly treat NSCs. To date, this approach has mostly been used in toxin studies [136, 137], yet it offers a promising avenue to analyze how stress and inflammatory insults impact placental transport and metabolism, and the direct subsequent effects this may have on NSCs. Additional studies should also test the effects of sex hormones in this context.

## Conclusions

In conclusion, this is the first systematic review reporting on the effect of cortisol and inflammatory cytokines on placental cells and foetal brain cells in vitro. Overall, our findings indicate that stress and inflammation not only negatively affect the placenta’s regulation of nutrients and hormones to the foetus but also trigger downstream pathways, resulting in adverse effects on foetal brain development due to heightened inflammation in the placenta and increased transport of cortisol to the foetus. These effects are mediated by a variety of cellular, molecular, and epigenetic mechanisms. Future studies should further investigate the role of sex hormones in these processes, and advance in vitro models by incorporating both placental and NPCs using innovative methodologies. Such advancements may eventually contribute to the development of more targeted therapeutic approaches.

## Supplementary information

PRISMA_2020_checklist
